# Increased prevalence and severity of femoral bone wear in Japanese patients with varus knee osteoarthritis

**DOI:** 10.1002/ksa.12697

**Published:** 2025-05-12

**Authors:** Manabu Akagawa, Hidetomo Saito, Yasuhiro Takahashi, Hiroaki Kijima, Yuji Kasukawa, Koji Nozaka, Naohisa Miyakoshi

**Affiliations:** ^1^ Department of Orthopedic Surgery Akita University Graduate School of Medicine Akita Japan; ^2^ Department of Orthopedic Surgery Omagari Kousei Medical Center Daisen Akita Japan

**Keywords:** bone wear, femoral condyle, kinematic alignment total knee arthroplasty, osteoarthritis

## Abstract

**Purpose:**

The calliper technique in kinematic alignment total knee arthroplasty assumes nearly identical medial and lateral femoral condylar radii and negligible subchondral bone wear. However, racial differences were not considered. This study aimed to assess the degree and severity of bone wear in Japanese patients with varus knee osteoarthritis.

**Methods:**

This cross‐sectional study included 155 knees from 130 patients who underwent total or unicompartmental knee arthroplasty for primary varus‐type knee osteoarthritis between April 2020 and March 2024. Preoperative computed tomography scans were used to measure the femoral condylar radii via a circle‐fitting technique. Bone wear was assessed at 0° and 90° by measuring the distance between the best‐fit circle and the subchondral bone periphery, with additional measurements at the peak wear angle, if present.

**Results:**

Among 155 knees, 16 (10.3%) exhibited bone wear. Bone wear >1 mm at 0° was observed in 3.2% of the cases. The peak wear angle was 43.1 ± 5.0°, with a mean depth of 2.0 ± 0.7 mm. The medial and lateral condylar radii were nearly identical (18.3 ± 1.2 mm vs. 18.2 ± 1.2 mm, *p* = 0.002), with a strong correlation (*R* ^2^ = 0.94, *p* < 0.001).

**Conclusion:**

Bone wear exceeding 1 mm at 0° was observed in 3.2% of cases, while overall bone wear was present in 10.3% of knees. Although the difference in radii between the medial and lateral femoral condyles was statistically significant, it was clinically negligible. This finding supports the reliability of the cylindrical axis as a reference for surgical techniques. These results highlight the importance of recognizing potential racial differences in bone wear and underscore the need for accurate assessment to achieve anatomic restoration in kinematic alignment total knee arthroplasty.

**Level of Evidence:**

Level III.

**Abbreviations**
CPAK: coronal plane alignment of the knee
CT: computed tomography
FKP: functional knee phenotype
FMA: femoral mechanical angle
HKA: hip–knee–ankle angle
KA: kinematic alignment
LDFA: lateral distal femoral angle
MPTA: medial proximal tibial angle
MRI: magnetic resonance imaging
NEU: neutral
OA: osteoarthritis
TKA: total knee arthroplasty
TMA: tibial mechanical angle
UKA: unicompartmental knee arthroplasty
VAL: valgus
VAR: varus

## INTRODUCTION

Kinematic alignment total knee arthroplasty (KA‐TKA) aims to restore the native knee anatomy by reconstructing the three key axes of the knee [17, 28]. This technique has been reported to achieve favourable clinical outcomes [2, 4, 5, 32, 36], long‐term results [15] and biomechanical advantages [1, 19, 35] compared to conventional alignment methods.

The calliper technique [14], a fundamental technique of KA‐TKA, assumes that the medial and lateral femoral condyles have nearly identical radii [16] and that femoral subchondral bone wear is negligible [27]. Therefore, this technique was performed without accounting for bone wear. This technique has been shown to enable highly reproducible reconstruction of the cylindrical axis [33], the flexion–extension axis of the tibiofemoral joint [39]. However, this approach does not consider racial differences. Japanese individuals are known to have distinct bone morphology [26] and a greater degree of varus alignment than other populations [38]. Furthermore, femoral bowing deformity has been reported to progress with osteoarthritis (OA) [24], and could potentially contribute to changes in the radii of the medial and lateral femoral condyles. Given the stronger varus alignment in Japanese patients, bone wear may be more frequent and severe.

Therefore, the primary objective of this study was to investigate the prevalence and severity of bone wear in Japanese populations with varus knee OA. The secondary objective was to evaluate the radii of the medial and lateral femoral condyles. Given the greater degree of varus alignment in this population, we hypothesized that bone wear is more frequent and severe in the Japanese population. We also hypothesized that OA‐related changes contribute to differences in the radii of the medial and lateral femoral condyles.

## MATERIALS AND METHODS

### Patients

This cross‐sectional study included 155 knees from 130 patients who underwent total or unicompartmental knee arthroplasty (UKA) for primary varus‐type knee OA between April 2020 and March 2024. Written informed consent was obtained from all patients, and the hospital's ethics committee approved this study (approval no. # 23‐022).

### Bone wear and condylar radii analysis

**Figure 1:**
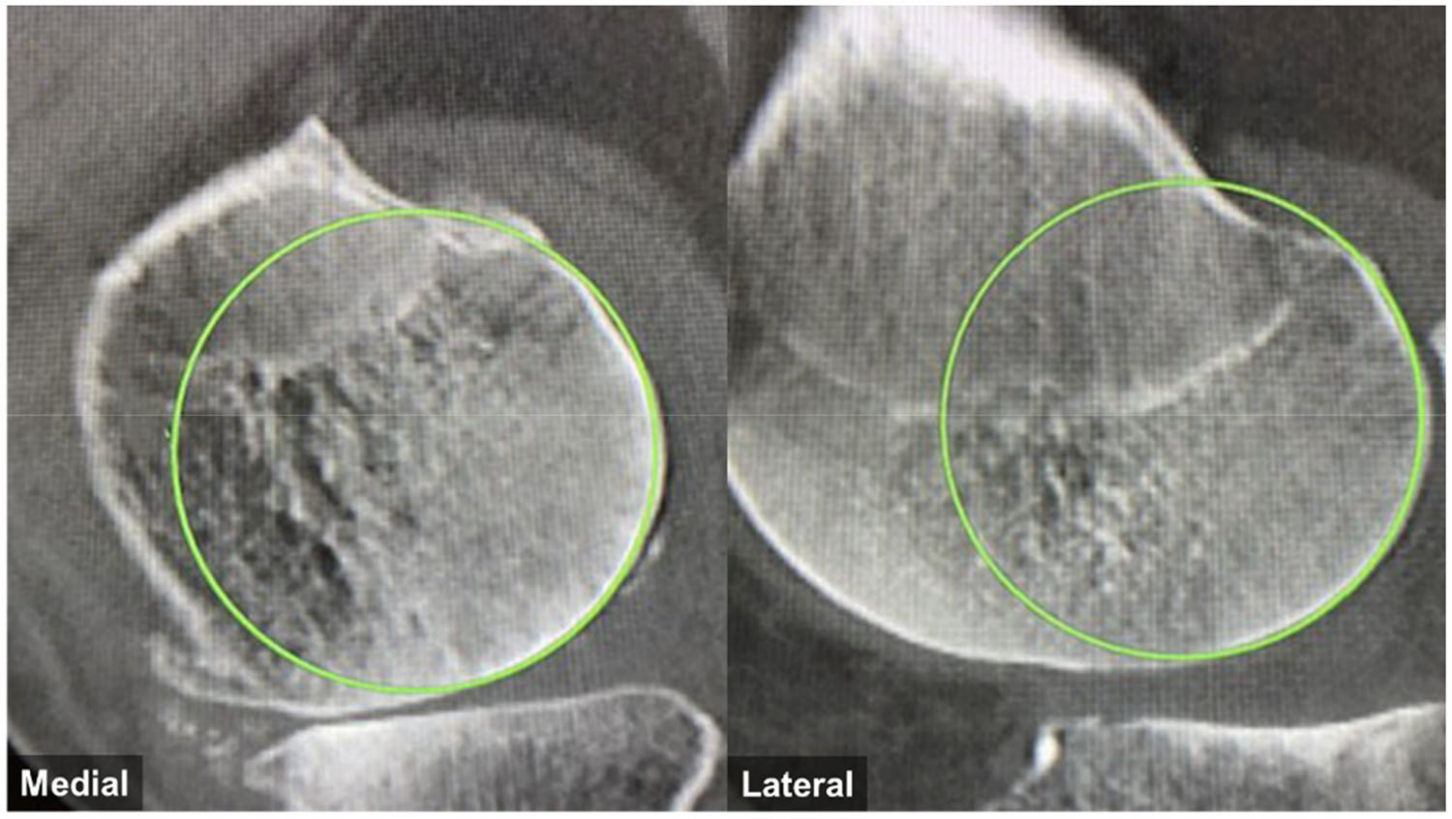
Assessment of the radii of medial and lateral femoral condyles. The radii of the medial and lateral femoral condyles were independently measured using a circle‐fitting technique.

**Figure 2:**
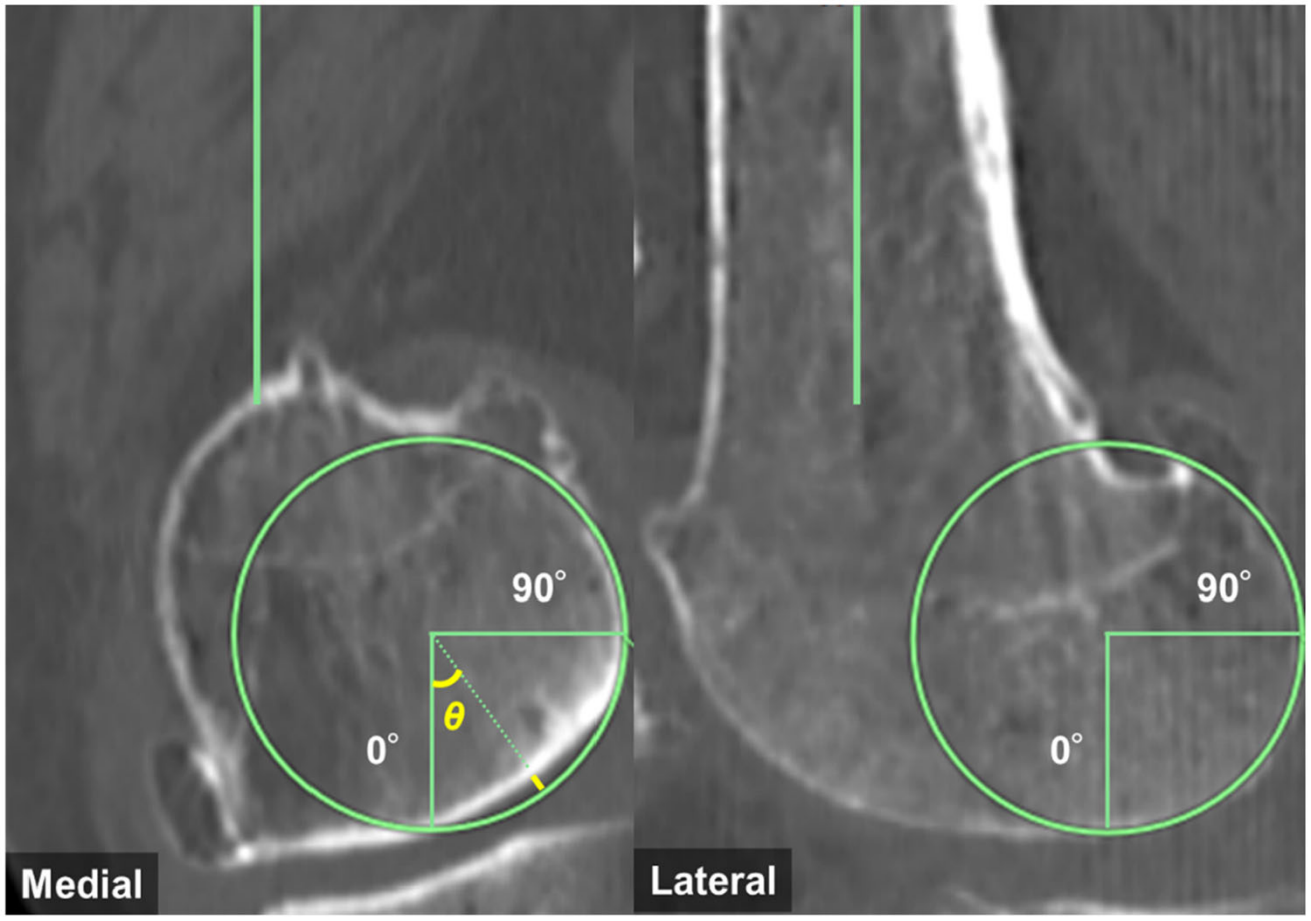
Measurement of bone wear in the femoral condyles. Bone wear was assessed by measuring the distance between the best‐fit circle and the subchondral bone periphery at 0° and 90°, with additional measurement at the peak wear angle (*θ*) if present. (The yellow line indicates bone wear, and the yellow angle represents *θ*.)

Preoperative computed tomography (CT) scans from the pelvis to the ankle, with 1 mm increments, were used to evaluate the femoral condyles (Siemens SOMATOM Definition AS syngo CT VB20). According to previous reports [16, 29], the radii of the medial and lateral femoral condyles were independently measured within the 10° to 160° range using a circle‐fitting technique (Figure 1). Radii were determined by fitting a best‐fit circle to four consecutive images showing the greatest curvature of the femoral condyle. The average radius obtained from these images was as the condylar radius. To minimize the influence of bone wear, regions without subchondral sclerosis were selected as reference points for the circle‐fitting technique. Cases were considered to have bone wear if the wear exceeded 0.5 mm, as smaller values could fall within the range of measurement error. These cases were excluded to ensure the accuracy of the radius measurement. The average radii of the medial and lateral femoral condyles were calculated and compared. In cases of bone wear, the best‐fit circle from the unworn condyle was copied and aligned to the subchondral bone boundary of the worn condyle to assess the amount of bone wear [27]. Bone wear was evaluated by measuring the distance between the best‐fit circle and the subchondral bone periphery at 0° and 90°. Additionally, if the maximum bone wear occurred at an angle other than 0° or 90°, its angle (*θ*) and extent were also measured (Figure 2). All radiographic measurements were performed using the PACS software package (SYNAPSE 5, FUJIFILM Medical Co. Ltd.).

To assess measurement reliability, intra‐ and inter‐observer reliabilities were evaluated using 15 randomly selected knees. For the medial condyle, the intra‐ and inter‐observer reliability were 0.93 and 0.92, respectively, while for the lateral condyle, they were 0.89 and 0.92, respectively. These results indicate good‐to‐excellent reliability.

### Alignment assessment and classification

Full‐length standing radiographs were used to evaluate lower limb alignment. Of the 155 knees initially assessed, 3 were excluded due to inadequate imaging, resulting in 152 knees being included in the final analysis.

The hip–knee–ankle angle (HKA) was defined as the angle between the mechanical axes of the femur and tibia. The femoral mechanical angle (FMA) was defined as the medial angle between the femoral mechanical axis and the distal femoral joint line, whereas the lateral distal femoral angle (LDFA) represented the corresponding lateral angle. The tibial mechanical angle (TMA), equivalent to the medial proximal tibial angle (MPTA), was defined as the medial angle between the tibial mechanical axis and the proximal tibial joint line. Arithmetic HKA (aHKA) was calculated as MPTA minus LDFA, while joint line obliquity (JLO) was calculated as their sum. The functional knee phenotype (FKP) was determined by classifying each knee based on HKA, FMA and TMA into predefined categories and expressed as a composite label (e.g., ‘NEU_HKA_0° + NEU_FMA_0° + NEU_TMA_0°’), as previously described [12]. The coronal plane alignment of the knee (CPAK) classification was based on a combination of aHKA (varus < –2°, neutral –2° to +2°, valgus > +2°) and JLO (apex distal < 177°, neutral 177°–183°, apex proximal > 183°), resulting in nine types (Types I–IX), according to the previous report [22].

### Statistical analysis

All measurements were normally distributed, and all results were expressed as mean ± standard deviation. A paired *t* test was used to determine whether the radii of the medial and lateral femoral condyles differed. Simple regression analysis was performed to assess the strength of the association between the radii of the medial and lateral condyles.

G*Power analysis (Ver.3.1.9.6) showed that 34 knees (*α* = 0.05, 1 − *β* = 0.8, effect size = 0.5) for a paired *t* test and 55 knees (*α* = 0.05, 1 − *β* = 0.8, effect size = 0.15) for the simple regression analysis were necessary to achieve statistical power.

All statistical analyses were performed using EZR [18], and statistical significance was set at *p* < 0.05.

## RESULTS

**Figure 3:**
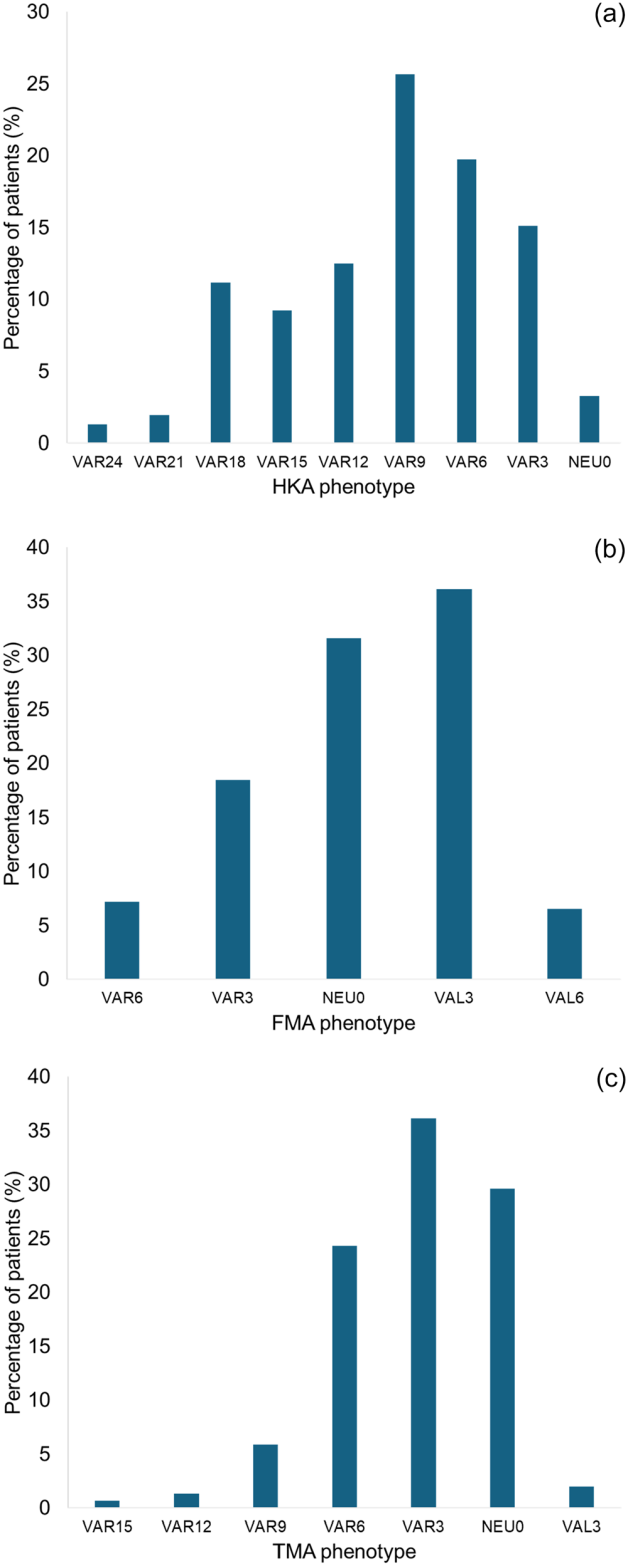
The distributions of hip–knee–ankle angle (HKA) (a), femoral mechanical angle (FMA) (b) and tibial mechanical angle (TMA) (c) phenotypes. These histograms illustrate the distribution of each alignment parameter that defines FKP. HKA phenotypes ranged widely, including severe varus alignment (a). FMA phenotypes were concentrated in the neutral to mild valgus range (b), whereas TMA phenotypes showed greater variability within the varus spectrum (c).

Patient demographic variables, alignment characteristics and the three most common CPAK classifications, along with the four most common FKP types, are summarized in Table 1. The distributions of the HKA, FMA, and TMA phenotypes are presented in Figure 3.

**Table 1:** Patient characteristics.

Patients (*n*)		130
Knees (*n*)		155
Age (years)		76.2 (7.2)
Sex (knees)	Male	33
	Female	122
Height (cm)		152.1 (8.2)
BMI (kg/m^2^)		25.9 (3.9)
Kellgren–Lawrence grade (knees)	III	26
	IV	129
Alignment parameters	mHKA (°)	−9.9 (5.3)
	LDFA (°)	89.5 (3.0)
	MPTA (°)	83.8 (3.0)
CPAK classification (knees, %)	Type I	102 (65.8%)
	Type II	22 (14.2%)
	Type IV	20 (12.9%)
Functional knee phenotype (knees, %) (common 4 types)	VAR_HKA_9° NEU_FMA_0° VAR_TMA_3°	10 (6.6%)
	VAR_HKA_3° VAL_FMA_3° NEU_TMA_0°	10 (6.6%)
	VAR_HKA_6° VAL_FMA_3° VAR_TMA_3°	8 (5.3%)
	VAR_HKA_6° NEU_FMA_0° NEU_TMA_0°	8 (5.3%)

Abbreviations: BMI, body mass index; CPAK, coronal plane alignment of the knee; FMA, femoral mechanical angle; LDFA, lateral distal femoral angle; mHKA, mechanical hip–knee–ankle angle; MPTA, medial proximal tibial angle; NEU, neutral; TMA, tibial mechanical angle; VAL, valgus; VAR, varus.

**Figure 4:**
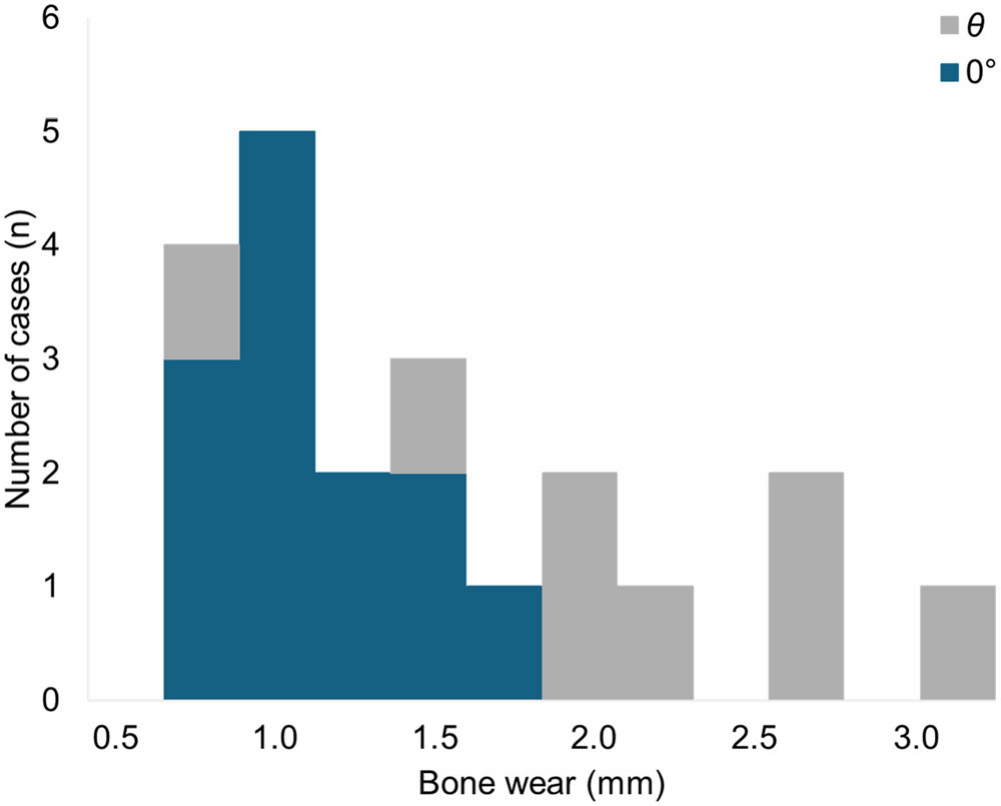
Distribution of bone wear depths at 0° and *θ.* Bone wear was predominantly observed at *θ* (43.1 ± 5.0°), with an average depth of 2.0 ± 0.7 mm, showing a tendency for greater wear at *θ* compared to 0°.

Of the 155 knees analyzed, 16 knees (10.3%) exhibited bone wear. The bone wear findings are summarized in Table 2. No bone wear was observed at 90° in the medial condyle or at either 0° or 90° in the lateral condyle. In the medial condyle, the average wear depth at 0° was 1.0 ± 0.3 mm, and wear exceeding 1 mm was observed in five knees (3.2%). At angle *θ* (43.1 ± 5.0°), the average wear depth was 2.0 ± 0.7 mm, with wear exceeding 1 mm observed in eight knees (5.2%). The distribution of wear depths is shown in Figure 4. The post hoc sample size calculation for the proportion of knees with bone wear was performed using the observed prevalence of 10.3%. With a confidence interval of ±5%, the required sample size was determined to be 142 knees, confirming that this study included enough cases.

**Table 2:** Bone wear analysis of 16 knees.

Location	Angle (°)	Bone wear (mm)	Range (mm)	Wear > 1 mm (*n*)
Medial	0	1.0 (0.3)	0.5–1.5	5
	*θ*: 43.1 (5.0)	2.0 (0.7)	0.7–3.1	8
	90	–	–	–
Lateral	0	–	–	–
	90	–	–	–

**Figure 5:**
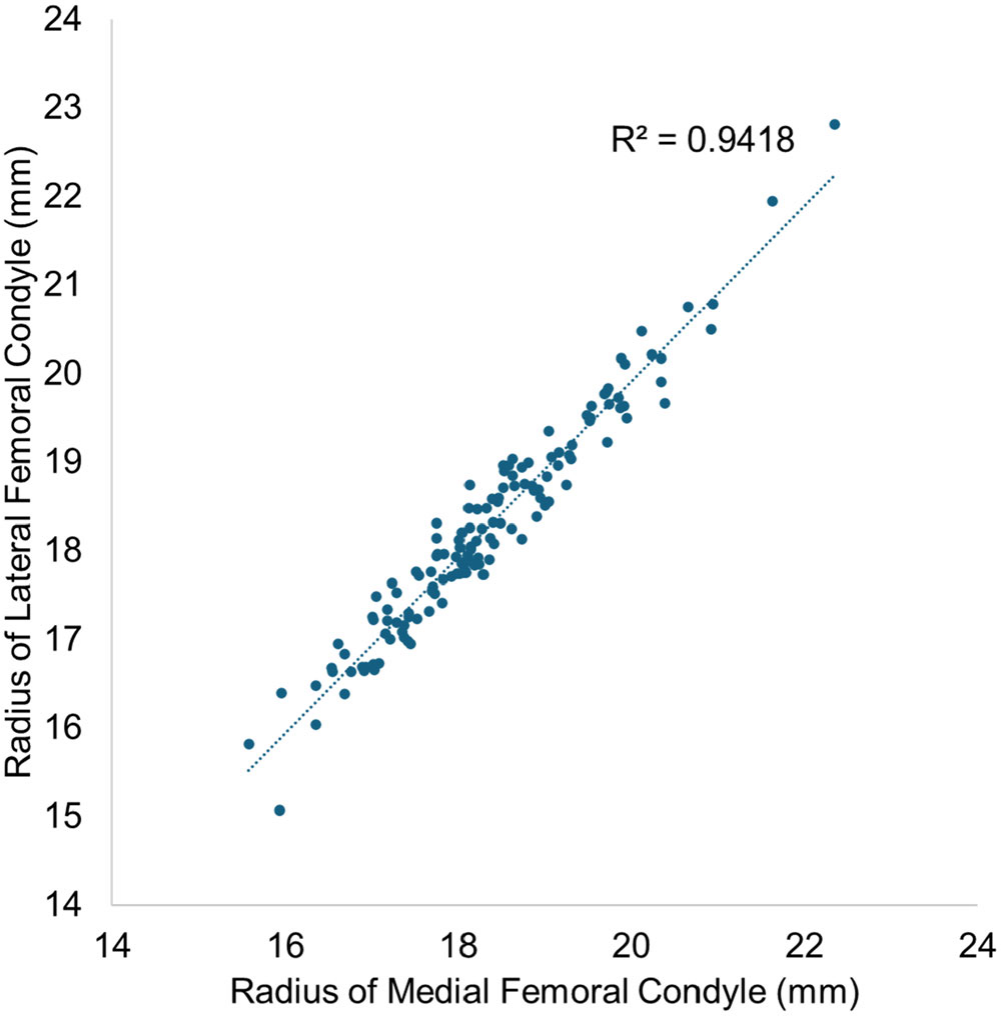
The associations between the radii of the medial and lateral femoral condyles. A strong positive correlation was observed between the radii of the medial and lateral femoral condyles.

The medial condyle radius was 0.1 mm larger than the lateral condyle, with a statistically significant difference (18.3 ± 1.2 mm vs 18.2 ± 1.2 mm, *p* = 0.002). A strong positive correlation was observed between the radii of medial and lateral condyles (*R* ^2^ = 0.94, *p* < 0.001, Figure 5).

## DISCUSSION

The most important finding of this study was that bone wear exceeding 1 mm at 0° in the medial femoral condyle was observed in 3.2% of the cases, with overall bone wear present in 10.3% of the cases across all locations. To the best of our knowledge, this is the first report to evaluate the frequency and severity of bone wear in a Japanese population. Additionally, the radii of the medial and lateral femoral condyles differed by a statistically significant 0.1 mm, a difference considered clinically negligible. This suggests that the femoral condyles maintained a largely symmetrical morphology.

Few detailed reports exist on the extent of femoral bone wear in knees with OA. In the Japanese population, Hiranaka et al. compared the LDFA between knees with medial OA and contralateral non‐OA knees, and found a significant varus difference of 1.0° in OA knees, indicating the presence of bone wear [10]. They cited an Oxford UKA case in which a gap between the femoral component and bone was observed as an example of bone wear. In our results, bone wear was greater at an average angle of 43.1° than at 0°, suggesting that wear in this region may contribute to the gap between the component and bone. In comparison, Nam et al. reported that in varus knees, bone wear exceeding 1 mm was observed in only 0.2% of cases [27], whereas our study found a higher incidence of 3.2% at 0°. When considering all observed locations, bone wear was present in 10.3% of the cases. These findings suggest that Japanese individuals have a higher frequency of bone wear.

In our study, most knees were classified as CPAK Type I (65.8%), consistent with a previous report [37], showing a higher prevalence of this type in Japanese OA knees compared to 16.7%–33.4% in other countries [22, 23, 34]. FKP allows a more detailed evaluation of coronal alignment, indicating a more pronounced varus deformity, with lower mean and minimum HKA values than those reported in Western populations [3, 11]. This greater severity of varus alignment may have contributed to the higher prevalence of bone wear observed in our study. Our findings highlight ethnic differences in the pattern and extent of bone wear, which may have important implications for KA‐TKA. Unrecognized bone loss can lead to joint line elevation and mid‐flexion laxity [21], underscoring the need for careful preoperative assessment of bone wear. Intraoperative evaluation of cartilage thickness reportedly improves femoral reconstruction in TKA [6], and phenotype‐based alignment strategies can help develop a more precise surgical plan [7]. Recent advances in these approaches have improved the accuracy of anatomical reconstruction, and incorporating bone wear assessment may further refine surgical precision.

Several studies have investigated the radii of the femoral condyles (Table 3). In healthy knees, previous studies have shown that the medial and lateral femoral condyles are nearly identical in size, with minimal or no significant differences in radii [13, 25, 40]. Cadaveric studies have reported similar findings, confirming condylar symmetry [20, 30]. In OA knees, a previous report showed that the lateral condyle was slightly larger than the medial condyle in varus knees, with an average difference of 0.1 mm [16]. Another study found no significant average difference but noted that in 46 of 122 knees, the condylar radii differed by more than 1 mm [29]. In our study, cases with a difference in condylar radii greater than 1 mm were rare, suggesting that discrepancies in previous reports may be due to measurements that included worn regions. To minimize this effect, we excluded knees with bone wear and selected preserved areas without subchondral sclerosis. Even in worn knees, when referencing these preserved regions, the radii remained similar (18.4 ± 0.9 mm vs. 18.3 ± 0.8 mm), resulting in consistent and reliable measurements. Although OA progression is associated with various morphological changes, including reduced femoral anteversion, altered condylar orientation, increased tibial slope, and rotational malalignment [8, 9, 24, 31], our findings are consistent with those of previous studies on healthy, cadaveric, and OA knees, demonstrating that the medial and lateral femoral condylar radii remain nearly identical even in advanced OA. This preservation supports the cylindrical axis as a reliable reference for KA‐TKA.

**Table 3:** Summary of previous reports regarding the radii of femoral condyle in varus knee.

Authors	Participants	Methods	Medial condyle (mm)	Lateral condyle (mm)
Yue et al. [40]	Healthy volunteer	CT	17.3 ± 1.6	18.1 ± 2.0
Monk et al. [25]	Healthy volunteer	MRI	21 ± 1.8	21 ± 2.0
Hokari et al. [13]	Healthy volunteer	CT	17.0 ± 1.6	17.1 ± 1.8
Lustig et al. [20]	Cadaver	CT	17.8 ± 1.4	17.8 ± 1.9
Pinskerova et al. [30]	Cadaver	MRI	22	21
Howell et al. [16]	Patients with OA	MRI	19.4 ± 2.0	19.5 ± 2.0
Niki et al. [29]	Patients with OA	CT	17.4 ± 1.6	17.3 ± 1.4
Present study	Patients with OA	CT	18.3 ± 1.2	18.2 ± 1.2

Abbreviations: CT, computed tomography; MRI, magnetic resonance imaging; OA, osteoarthritis.

Furthermore, the medial and lateral femoral condylar radii observed in our study were consistent with previous reports in the Japanese population [13, 29], whereas studies in Western populations have reported larger values [16, 25, 30]. These anatomical differences are clinically relevant in TKA, where the use of implants designed based on Western morphology may result in the posterior condylar overhang. This highlights the potential need for population‐specific implant designs tailored to the Japanese anatomical profile.

This study has several limitations. First, only varus knees were included, as the number of valgus knees in our cohort was too small for statistical analysis. Consequently, potential differences between varus and valgus knees were not assessed, which may limit the generalizability of our findings. A larger cohort including valgus knees is required to provide a more comprehensive understanding. Second, the relatively small sample size from a single institution may limit the statistical power and generalizability of the results. Although the sample size was sufficient to detect differences in femoral condylar radii and bone wear prevalence, the limited number of cases with bone wear (*n* = 16) precluded subgroup or multivariate analyses. Larger studies are required to validate these findings and investigate the influence of potential confounding factors such as age, BMI, and OA severity. Finally, the 0° flexion position lies outside the 10–160° circle‐fitting range, and the applied method may not fully capture the femoral geometry at this angle. However, we adopted the same evaluation method as in a previous study [27] to enable meaningful comparisons with Western populations. Future studies employing alternative approaches may better elucidate the distribution of bone wear at 0° flexion.

In conclusion, we investigated the prevalence and severity of bone wear in Japanese patients with varus knee OA. Bone wear exceeding 1 mm at 0° was observed in 3.2% of cases, and overall bone wear was present in 10.3% of the cases. In addition, the medial and lateral femoral condyles were nearly identical in size, with a statistically significant but clinically negligible difference. These findings provide novel insights into racial differences in bone wear and may contribute to more accurate restoration of the native knee anatomy in KA‐TKA.

## AUTHOR CONTRIBUTIONS

**Manabu Akagawa**: Conceptualization; methodology; formal analysis; investigation; writing—original draft preparation. **Hidetomo Saito**: Conceptualization; methodology. **Yasuhiro Takahashi**: Methodology. **Yuji Kasukawa**: Writing—review and editing. **Koji Nozaka**: Writing—review and editing. **Naohisa Miyakoshi**: Supervision.

## CONFLICT OF INTEREST STATEMENT

The authors declare no conflicts of interest.

## ETHICS STATEMENT

The local ethics committee of the hospital approved this study (approval no. 23‐022). The authors certify that the study was performed in accordance with the ethical standards as laid down in the 1964 Declaration of Helsinki and its later amendments. Informed consent was obtained from all individual participants included in the study.

## Data Availability

The data that support the findings of this study are available on request from the corresponding author. The data are not publicly available due to privacy or ethical restrictions.

## References

[1] Blakeney W , Clément J , Desmeules F , Hagemeister N , Rivière C , Vendittoli P‐A . Kinematic alignment in total knee arthroplasty better reproduces normal gait than mechanical alignment. Knee Surg Sports Traumatol Arthrosc. 2019;27:1410–1417. 30276435 10.1007/s00167-018-5174-1

[2] Calliess T , Bauer K , Stukenborg‐Colsman C , Windhagen H , Budde S , Ettinger M . PSI kinematic versus non‐PSI mechanical alignment in total knee arthroplasty: a prospective, randomized study. Knee Surg Sports Traumatol Arthrosc. 2017;25:1743–1748. 27120192 10.1007/s00167-016-4136-8

[3] Chelli S , Rudyy T , Avram GM , Huegli RW , Amsler F , Hirschmann MT . Gender‐based differences exist in the functional knee phenotypes classification of the osteoarthritic knee. Knee Surg Sports Traumatol Arthrosc. 2024;32:2505–2515. 38415864 10.1002/ksa.12082

[4] Courtney PM , Lee G‐C . Early outcomes of kinematic alignment in primary total knee arthroplasty: a meta‐analysis of the literature. J Arthroplasty. 2017;32:2028–2032. 28341278 10.1016/j.arth.2017.02.041

[5] Dossett HG , Estrada NA , Swartz GJ , LeFevre GW , Kwasman BG . A randomised controlled trial of kinematically and mechanically aligned total knee replacements: two‐year clinical results. Bone Joint J. 2014;96–B:907–913. 10.1302/0301-620X.96B7.32812 24986944

[6] Giurazza G , Campi S , Hirschmann MT , Franceschetti E , Tanzilli A , Gregori P , et al. Cartilage thickness can be accurately measured intraoperatively in total knee arthroplasty: a step further in calipered kinematic alignment. J Exp Orthop. 2025;12(1):e70155. 39867675 10.1002/jeo2.70155 PMC11763056

[7] Graichen H , Avram GM , Strauch M , Kaufmann V , Hirschmann MT . Tibia‐first, gap‐balanced patient‐specific alignment restores bony phenotypes and joint line obliquity in a great majority of varus and straight knees and normalises valgus and severe varus deformities. Knee Surg Sports Traumatol Arthrosc. 2024;32:1287–1297. 38504509 10.1002/ksa.12145

[8] Hess S , Moser LB , Robertson EL , Behrend H , Amsler F , Iordache E , et al. Osteoarthritic and non‐osteoarthritic patients show comparable coronal knee joint line orientations in a cross‐sectional study based on 3D reconstructed CT images. Knee Surg Sports Traumatol Arthrosc. 2022;30:407–418. 34564737 10.1007/s00167-021-06740-3 PMC8866364

[9] Hess S , Sabrina C , Leclercq V , Lustig S , Graichen H , Hirschmann MT . Three‐compartment phenotype concept of total knee arthroplasty alignment: mismatch between distal femoral, posterior femoral, and tibial joint lines. J Arthroplasty. 2025;40:2023–2034. 10.1016/j.arth.2025.02.015 40049560

[10] Hiranaka T , Fukai Y , Tanaka S , Okajima T , Ishida Y , Koide M , et al. Bone loss on the femoral side in knees with medial osteoarthritis: implications for kinematically‐aligned total knee arthroplasty—a comparative study of lateral distal femoral angle in knees with and without osteoarthritis in the same patients. Knee. 2024;49:62–69. 38870616 10.1016/j.knee.2024.05.002

[11] Hirschmann MT , Khan ZA , Sava MP , von Eisenhart‐Rothe R , Graichen H , Vendittoli P‐A , et al. Definition of normal, neutral, deviant and aberrant coronal knee alignment for total knee arthroplasty. Knee Surg Sports Traumatol Arthrosc. 2024;32:473–489. 38293728 10.1002/ksa.12066

[12] Hirschmann MT , Moser LB , Amsler F , Behrend H , Leclerq V , Hess S . Functional knee phenotypes: a novel classification for phenotyping the coronal lower limb alignment based on the native alignment in young non‐osteoarthritic patients. Knee Surg Sports Traumatol Arthrosc. 2019;27:1394–1402. 30976825 10.1007/s00167-019-05509-z

[13] Hokari S , Tanifuji O , Kobayashi K , Mochizuki T , Katsumi R , Sato T , et al. The inclination of the femoral medial posterior condyle was almost vertical and that of the lateral was tilted medially. Knee Surg Sports Traumatol Arthrosc. 2020;28:3858–3864. 32016580 10.1007/s00167-020-05856-2

[14] Howell SM . Calipered kinematically aligned total knee arthroplasty: an accurate technique that improves patient outcomes and implant survival. Orthopedics. 2019;42:126–135. 31099877 10.3928/01477447-20190424-02

[15] Howell SM , Akhtar M , Nedopil AJ , Hull ML . Reoperation, implant survival, and clinical outcome after kinematically aligned total knee arthroplasty: a concise clinical follow‐up at 16 years. J Arthroplasty. 2024;39:695–700. 37659680 10.1016/j.arth.2023.08.080

[16] Howell SM , Howell SJ , Hull ML . Assessment of the radii of the medial and lateral femoral condyles in varus and valgus knees with osteoarthritis. J Bone Joint Surg Am. 2010;92:98–104. 10.2106/JBJS.H.01566 20048101

[17] Howell SM , Papadopoulos S , Kuznik KT , Hull ML . Accurate alignment and high function after kinematically aligned TKA performed with generic instruments. Knee Surg Sports Traumatol Arthrosc. 2013;21:2271–2280. 23948721 10.1007/s00167-013-2621-x

[18] Kanda Y . Investigation of the freely available easy‐to‐use software “EZR” for medical statistics. Bone Marrow Transplant. 2013;48:452–458. 23208313 10.1038/bmt.2012.244 PMC3590441

[19] Kang K‐T , Koh Y‐G , Nam JH , Kwon SK , Park KK . Kinematic alignment in cruciate retaining implants improves the biomechanical function in total knee arthroplasty during gait and deep knee bend. J Knee Surg. 2020;33:284–293. 30727015 10.1055/s-0039-1677846

[20] Lustig S , Lavoie F , Selmi TA , Servien E , Neyret P . Relationship between the surgical epicondylar axis and the articular surface of the distal femur: an anatomic study. Knee Surg Sports Traumatol Arthrosc. 2008;16:674–682. 18478201 10.1007/s00167-008-0551-9

[21] Luyckx T , Vandenneucker H , Ing LS , Vereecke E , Ing AV , Victor J . Raising the joint line in tka is associated with mid‐flexion laxity: a study in cadaver knees. Clin Orthop Relat Res. 2018;476:601–611. 29443845 10.1007/s11999.0000000000000067 PMC6260050

[22] MacDessi SJ , Griffiths‐Jones W , Harris IA , Bellemans J , Chen DB . Coronal Plane Alignment of the Knee (CPAK) classification. Bone Joint J. 2021;103–B:329–337. 10.1302/0301-620X.103B2.BJJ-2020-1050.R1 PMC7954147 33517740

[23] MacDessi SJ , Griffiths‐Jones W , Harris IA , Bellemans J , Chen DB . The arithmetic HKA (aHKA) predicts the constitutional alignment of the arthritic knee compared to the normal contralateral knee: a matched‐pairs radiographic study. Bone Jt Open. 2020;1:339–345. 33215122 10.1302/2633-1462.17.BJO-2020-0037.R1 PMC7659698

[24] Matsumoto T , Hashimura M , Takayama K , Ishida K , Kawakami Y , Matsuzaki T , et al. A radiographic analysis of alignment of the lower extremities—initiation and progression of varus‐type knee osteoarthritis. Osteoarthritis Cartilage. 2015;23:217–223. 25481289 10.1016/j.joca.2014.11.015

[25] Monk AP , Choji K , O'connor JJ , Goodfellow JW , Murray DW . The shape of the distal femur: a geometrical study using MRI. Bone Joint J. 2014;96–B:1623–1630. 10.1302/0301-620X.96B12.33964 25452364

[26] Moser LB , Hess S , de Villeneuve Bargemon J‐B , Faizan A , LiArno S , Amsler F , et al. Ethnical differences in knee phenotypes indicate the need for a more individualized approach in knee arthroplasty: a comparison of 80 asian knees with 308 caucasian knees. J Pers Med. 2022;12:121. 35055436 10.3390/jpm12010121 PMC8779125

[27] Nam D , Lin KM , Howell SM , Hull ML . Femoral bone and cartilage wear is predictable at 0° and 90° in the osteoarthritic knee treated with total knee arthroplasty. Knee Surg Sports Traumatol Arthrosc. 2014;22:2975–2981. 24839078 10.1007/s00167-014-3080-8

[28] Nedopil AJ , Howell SM , Hull ML , Hirschmann MT . A TKA can be kinematically aligned without restrictions: current evidence. Knee Surg Sports Traumatol Arthrosc. 2024;32:1354–1358. 38501289 10.1002/ksa.12132

[29] Niki Y , Nagai K , Sassa T , Harato K , Suda Y . Comparison between cylindrical axis‐reference and articular surface‐reference femoral bone cut for total knee arthroplasty. Knee Surg Sports Traumatol Arthrosc. 2017;25:3741–3746. 27485125 10.1007/s00167-016-4251-6

[30] Pinskerova V , Iwaki H , Freeman MA . The shapes and relative movements of the femur and tibia at the knee. Orthopade. 2000;29(Suppl 1):3–5. 10.1007/pl00003679 10929342

[31] Puthumanapully PK , Harris SJ , Leong A , Cobb JP , Amis AA , Jeffers J . A morphometric study of normal and varus knees. Knee Surg Sports Traumatol Arthrosc. 2014;22:2891–2899. 25261224 10.1007/s00167-014-3337-2 PMC4237928

[32] Rivière C , Iranpour F , Auvinet E , Howell S , Vendittoli P‐A , Cobb J , et al. Alignment options for total knee arthroplasty: a systematic review. Orthop Traumatol Surg Res. 2017;103:1047–1056. 28864235 10.1016/j.otsr.2017.07.010

[33] Rivière C , Iranpour F , Harris S , Auvinet E , Aframian A , Chabrand P , et al. The kinematic alignment technique for TKA reliably aligns the femoral component with the cylindrical axis. Orthop Traumatol Surg Res. 2017;103:1069–1073. 28870873 10.1016/j.otsr.2017.06.016

[34] Sappey‐Marinier E , Batailler C , Swan J , Schmidt A , Cheze L , MacDessi SJ , et al. Mechanical alignment for primary TKA may change both knee phenotype and joint line obliquity without influencing clinical outcomes: a study comparing restored and unrestored joint line obliquity. Knee Surg Sports Traumatol Arthrosc. 2022;30:2806–2814. 34291311 10.1007/s00167-021-06674-w

[35] Schelker BL , Moret CS , Sava MP , von Eisenhart‐Rothe R , Graichen H , Arnold MP , et al. The impact of different alignment strategies on bone cuts in total knee arthroplasty for varus knee phenotypes. Knee Surg Sports Traumatol Arthrosc. 2023;31:1840–1850. 36811657 10.1007/s00167-023-07351-w PMC10089997

[36] Takahashi T , Ansari J , Pandit HG . Kinematically aligned total knee arthroplasty or mechanically aligned total knee arthroplasty. J Knee Surg. 2018;31:999–1006. 29444542 10.1055/s-0038-1632378

[37] Toyooka S , Osaki Y , Masuda H , Arai N , Miyamoto W , Ando S , et al. Distribution of coronal plane alignment of the knee classification in patients with knee osteoarthritis in Japan. J Knee Surg. 2023;36:738–743. 35114721 10.1055/s-0042-1742645

[38] Wanezaki Y , Suzuki A , Takakubo Y , Nakajima T , Toyono S , Toyoshima S , et al. Lower limb alignment in healthy Japanese adults. J Orthop Sci. 2023;28:200–203. 34815138 10.1016/j.jos.2021.10.016

[39] Yin L , Chen K , Guo L , Cheng L , Wang F , Yang L . Identifying the functional flexion‐extension axis of the knee: an in‐vivo kinematics study. PLoS One. 2015;10:0128877. 10.1371/journal.pone.0128877 PMC4454551 26039711

[40] Yue B , Varadarajan KM , Ai S , Tang T , Rubash HE , Li G . Gender differences in the knees of Chinese population. Knee Surg Sports Traumatol Arthrosc. 2011;19:80–88. 20407755 10.1007/s00167-010-1139-8

